# A comprehensive narrative review of scoliosis in children and adolescents: epidemiology, screening strategies, therapeutic interventions, and biomechanical research

**DOI:** 10.3389/fped.2026.1866246

**Published:** 2026-09-04

**Authors:** Sun Chuan, Cong Feina, Qiu Aigong, Xu Shaoling

**Affiliations:** 1 Binzhou Medical University, Binzhou, Shandong, China; 2 Zhaoyuan Hospital of Traditional Chinese Medicine, Yantai, Shandong, China

**Keywords:** adolescent idiopathic scoliosis, artificial intelligence, biomechanics, bracing, epidemiology, exercise therapy, mental health, screening strategies

## Abstract

Scoliosis in children and adolescents, particularly adolescent idiopathic scoliosis (AIS), is a complex three-dimensional spinal deformity with a well-defined global prevalence and a considerable disease burden. This narrative review synthesizes the latest advances in the field, encompassing epidemiology, screening strategies, conservative treatment (bracing and physiotherapeutic scoliosis-specific exercises), biomechanical research, and artificial intelligence applications. A recent global meta-analysis indicates that the overall prevalence of scoliosis in children and adolescents is approximately 1.65%, with a consistent female predominance observed across most studies (global pooled OR = 2.16, 95% CI: 2.01–2.33), although the magnitude of this sex disparity varies by region, and prevalence increasing with age. However, the included studies are geographically unbalanced, with limited data from low-latitude regions, which may bias the global prevalence estimate. School-based screening remains the cornerstone of early detection, but screening policies vary significantly across countries. The development of novel home-based screening tools offers a new approach to overcome the limitations of school screening, yet their widespread adoption is constrained by economic factors and parental awareness. Multiple randomized controlled trials and meta-analyses have confirmed the efficacy of bracing and physiotherapeutic scoliosis-specific exercises, but no unified clinical guidelines exist for selecting the optimal treatment strategy. Furthermore, long-term longitudinal evidence on the intrinsic link between treatment approaches and patients’ mental health is still lacking. In addition, biomechanical techniques such as finite element analysis provide new perspectives for elucidating the mechanical properties of scoliosis and optimizing treatment strategies, but most models are constructed under idealized conditions, differing from the physiological state, and their clinical translational value awaits further validation. This review integrates core evidence from the field, aiming to provide a reference for clinical practice and future research directions.

## 1 Introduction

### 1.1 Background and definition

Scoliosis is a complex three-dimensional spinal deformity diagnosed primarily by a lateral curvature of the spine in the coronal plane (Cobb angle ≥10°), accompanied by vertebral axial rotation and sagittal plane abnormalities (1). Idiopathic scoliosis is the most common clinical subtype, accounting for approximately 75%–85% of all cases, but its etiology remains incompletely understood (1, 2). Adolescent idiopathic scoliosis (AIS) specifically refers to idiopathic scoliosis diagnosed between the age of 10 years and skeletal maturity, representing the predominant clinical form of the disease (3, 4). The onset of AIS is closely associated with the adolescent growth spurt, and its etiology involves complex interactions among genetic, hormonal, biomechanical, and environmental factors (5, 6). However, existing studies have largely focused on single-factor analyses, and investigations into the synergistic pathogenetic mechanisms of multiple factors remain weak.

### 1.2 Disease burden and clinical significance

Beyond causing physical deformity, scoliosis can lead to pain, impaired cardiopulmonary function, functional limitations, and psychological issues such as anxiety, depression, and low self-esteem (1, 7, 8). A large database cohort study based on the Korean National Health Insurance Service showed that patients with AIS have a significantly higher risk of developing psychiatric disorders (e.g., mood disorders, anxiety disorders) compared with age-matched healthy controls, with adjusted odds ratios ranging from 1.5 to 1.7 (9). These findings underscore that mental health concerns should be regarded not merely as secondary consequences of the deformity but as integral components of the clinical presentation that warrant routine assessment and management. Although this study has a large sample size, it did not fully account for confounding factors like family environment and social support, and the generalizability of its conclusions to different ethnic and socioeconomic populations requires further verification. Clinically, untreated severe scoliosis (Cobb angle >50°) may continue to progress in adulthood, significantly reducing quality of life and often being accompanied by chronic pain (10, 11). Therefore, early detection and timely intervention are crucial for controlling disease progression and improving long-term outcomes.

Recent years have witnessed numerous breakthroughs in AIS research: large-scale epidemiological studies have updated prevalence data and risk factor information (1, 12); randomized controlled trials have clarified the clinical efficacy of bracing and scoliosis-specific exercises (13–15); biomechanical models such as finite element analysis have provided new tools for elucidating disease mechanisms and optimizing treatment planning (16–18); and artificial intelligence techniques are being progressively applied in screening, diagnosis, and prognosis prediction (19, 20). This article systematically integrates the latest evidence from these domains, aiming to provide a comprehensive reference for clinical decision-making and the identification of future research directions.

### 1.3 Aims of this review

Given the rapid accumulation of new evidence across multiple domains of scoliosis research, this narrative review aims to provide a comprehensive and up-to-date overview of the field. Specifically, this review addresses the following core questions:

1. What is the current global prevalence of scoliosis in children and adolescents, and what are the major risk factors associated with its onset?
2. What are the current screening strategies, what is their effectiveness, and how are novel home-based tools reshaping early detection?
3. What is the comparative evidence for conservative treatment modalities, including physiotherapeutic scoliosis-specific exercises (PSSE) and bracing, and how can they be optimally combined?
4. How do biomechanical studies, particularly finite element analysis, contribute to understanding scoliosis progression and optimizing treatment?
5. What is the role of artificial intelligence and machine learning in screening, diagnosis, and progression prediction, and what are the barriers to clinical translation?

By synthesizing evidence across these interconnected domains, this review aims to inform clinical decision-making and identify key gaps and priorities for future research.

## 2 Literature search strategy

This narrative review is based on a comprehensive but non-systematic literature search of peer-reviewed articles published between January 2015 and June 2026. The primary databases searched included PubMed, Web of Science, and the Cochrane Library. Search terms were combinations of the following keywords: “adolescent idiopathic scoliosis,” “AIS,” “scoliosis epidemiology,” “school screening,” “scoliosis screening,” “bracing,” “orthotic treatment,” “physiotherapeutic scoliosis-specific exercises,” “PSSE,” “Schroth,” “finite element analysis,” “spinal biomechanics,” “artificial intelligence,” and “machine learning.” Reference lists of retrieved articles and relevant review papers were also manually screened to identify additional eligible studies. Given the narrative scope of this review, priority was given to high-quality evidence including large-scale epidemiological studies, randomized controlled trials, meta-analyses, and recent prospective cohort studies. No formal quality assessment or quantitative synthesis was performed, consistent with the narrative review methodology.

## 3 Epidemiology and risk factors

### 3.1 Global prevalence

Accurate estimation of the prevalence of scoliosis in children and adolescents is a prerequisite for formulating public health policies and allocating medical resources efficiently. A large-scale systematic review and meta-analysis published in 2025, which included 239 studies with a total of 46,565,512 participants, provides the most comprehensive global prevalence data to date (1). The results showed that the global pooled prevalence of scoliosis in children and adolescents confirmed by radiography (Cobb angle ≥10°) was 1.65% (95% CI: 1.38–1.94), whereas the suspected prevalence based on a positive initial screening (e.g., positive Adam's forward bend test) was as high as 5.80% (95% CI: 4.83–6.86) (1). Notably, the considerable gap between positive screening rates and confirmed diagnosis rates is not only related to the sensitivity of initial screening methods but may also be influenced by the operational consistency of screeners, suggesting a need to optimize the screening process to improve efficiency.

Several factors may contribute to the observed gap between positive screening rates and confirmed radiographic diagnoses. First, not all children who screen positive undergo the recommended radiographic examination, either due to parental concerns about radiation exposure, financial constraints, or lack of access to healthcare facilities. Second, the Adam's forward bend test, the cornerstone of most school screening programs, is not exclusively sensitive to structural scoliosis; it can yield false-positive results in the presence of other conditions such as leg length discrepancy, pelvic obliquity, or asymmetrical paraspinal muscle tone, all of which may produce a visible trunk asymmetry without underlying vertebral rotation. Third, the operational consistency of screeners, particularly their training level and experience, can substantially influence the false-positive rate. These factors collectively contribute to the large gap between the 5.80% suspected prevalence and the 1.65% confirmed diagnosis rate reported in the global meta-analysis (1), and highlight the importance of incorporating more specific screening tools, such as scoliometer measurement or automated surface topography, as part of a two-step screening process to reduce unnecessary referrals. There are significant geographical differences in prevalence. According to the World Health Organization regional classification, the highest prevalence was observed in the European Region (2.88%), followed by the Region of the Americas (2.54%), with the lowest in the Eastern Mediterranean Region (1.22%); the prevalence in high-latitude areas (2.96%) was significantly higher than in mid- and low-latitude areas (1). The causes of these geographical differences remain inconclusive, being hypothesized to relate to genetic background, lifestyle habits, and climatic conditions, but targeted comparative studies to verify this are lacking.

Beyond the global meta-analysis, multiple region-specific studies have provided complementary prevalence data that further illustrate this geographical heterogeneity. According to the latest global meta-analysis by Wang et al. (2025), which included 239 studies with a total of 46.55 million participants, the prevalence of scoliosis in children and adolescents varied significantly by WHO region, with the highest rate observed in the European Region (2.88%), followed by the Region of the Americas (2.54%), and the lowest in the Eastern Mediterranean Region (1.22%) (1). In the United States, scoliosis affects approximately 2%–3% of the population, corresponding to an estimated 6 to 9 million individuals, with adolescent idiopathic scoliosis (AIS) being the most common form, affecting about 1%–3% of adolescents aged 10–16 years. A study from South America reported a total prevalence of 3.83% (95% CI: 2.74–4.92) among screened adolescents, with mild scoliosis accounting for 2.91%, moderate for 0.75%, and severe for 0.17% (21).

In Asia, prevalence figures vary considerably across countries and regions (22). A systematic review and meta-analysis of 42 studies involving 1,149,330 subjects from 30 regions in China reported a prevalence of 1.2% (95% CI: 1.1%–1.4%) among adolescents aged 10–18 years, with girls (1.6%) having a higher prevalence than boys (1.0%). Notably, this study also found that the prevalence increased from 0.9% during 2000–2015 to 1.6% during 2016–2024, suggesting a potential upward trend in recent years. Within China, significant regional variations exist (23):a large epidemiological study screening 139,922 multi-ethnic children in Dali, southwestern China, reported an overall prevalence of suspected scoliosis of 2.37%, with girls (2.5%) more affected than boys (2.0%) (24);another study in Yunnan Province reported a confirmed prevalence of 1.10% (0.87% in males and 1.32% in females) (25);while a study in Gansu Province, northwestern China, reported a higher suspected prevalence of 5.68%, with peak prevalence at 14 years for boys (6.70%) and 15 years for girls (8.75%) (26). In Singapore, the prevalence of AIS among schoolgirls is 1.37% at ages 11–12 years and increases to 2.22% at ages 13–14 years, with corresponding rates of 0.21% and 0.66% in boys (27). In Japan, recent large-scale school screening studies have suggested that the prevalence of AIS, generally estimated at approximately 2%–4%, may be increasing over time (28). In the Eastern Mediterranean Region, a cross-sectional study in Palestine reported that 5.4% of screened adolescents had AIS, highlighting the significant disease burden in this region.

Another systematic analysis of 2.22 million students in Mainland China reported a confirmed prevalence of 1.20%, with the risk for girls being approximately 1.57 times that for boys in this Chinese cohort (29). Although this study covers a large population, it did not thoroughly analyze how socioeconomic levels and differences in screening criteria contribute to regional prevalence variations.

The considerable variation in reported prevalence across studies—from as low as 0.47% to as high as 5.2%—likely reflects differences in screening methodologies, diagnostic criteria (suspected versus confirmed), population characteristics, and geographical factors, underscoring the need for standardized screening and reporting protocols to enable more meaningful cross-regional comparisons.

### 3.2 Age and sex distribution

Sex difference is the most prominent epidemiological feature of AIS. The aforementioned meta-analysis showed that the prevalence in girls (1.76%) was approximately twice that in boys (0.87%) (OR = 2.16, 95% CI: 2.01–2.33) (1), and this difference widens with age, becoming particularly pronounced during puberty. The core reason may be related to changes in female hormone levels and differences in skeletal growth rates, though the underlying mechanisms still require in-depth molecular biological investigation.

An additional contributing factor may relate to the temporal mismatch between the maturation of the extrapyramidal system—which is involved in posture control and motor coordination—and the onset of the adolescent growth spurt. In girls, the growth spurt typically begins around age 11, whereas extrapyramidal system maturation is not complete until approximately age 12; this two-year interval may create a window of vulnerability during which rapid skeletal growth outpaces neuromuscular control, potentially predisposing to the development of spinal deformity. In contrast, boys experience a more synchronized timeline, with the growth spurt starting around age 13, closer to the completion of extrapyramidal maturation. This developmental asynchrony may partly explain the marked female predominance observed in AIS, although direct evidence linking these temporal patterns to curve progression remains limited and warrants further investigation.

The prevalence increases continuously from the age of 7 years, peaking at 12–14 years in girls and 15–16 years in boys (1). The prevalence in adolescents (10–18 years; 1.13%) is approximately four times that in children (6–9 years; 0.26%) (1). This distribution pattern is closely associated with the adolescent growth spurt; however, there is currently a lack of quantitative analyses linking different growth rates to the risk of curve progression, which hinders precise screening of high-risk populations. Notably, the magnitude of the sex disparity in AIS prevalence varies considerably across studies. While the global meta-analysis reported an odds ratio of 2.16 for female sex as a risk factor (1), region-specific studies have reported more modest differences, such as the 1.57-fold higher risk observed in a large Chinese cohort (29). This variation likely reflects differences in study populations, screening methodologies, diagnostic criteria, and geographical or ethnic factors, rather than inconsistency in the underlying evidence. Specifically, the global estimate represents a pooled average across diverse populations, whereas region-specific estimates are influenced by local prevalence patterns and screening practices. These complementary perspectives collectively confirm a consistent female predominance in AIS, while highlighting the need for region-specific public health strategies and further investigation into the environmental and genetic determinants of this sex disparity.

### 3.3 Risk factors

The meta-analysis by Wang et al., based on 239 studies, identified multiple risk factors significantly associated with AIS, which can be divided into personal characteristics and behavioral habits (1). For personal characteristics, female sex (OR = 2.16), low body mass index (BMI, OR = 1.15), and family history (OR = 1.92) were all clear risk factors. Regarding behavioral habits, prolonged sitting (≥11 h/day, OR = 1.64), prolonged screen time (≥2 h/day, OR = 2.74), poor sitting posture (defined as habitual slouching, forward head position, or lateral trunk deviation during desk-based activities, OR = 3.48), unilateral physical activity (OR = 1.36), and insufficient outdoor activity time (<1 h/day, OR = 1.32) were all associated with the onset of AIS. Although this meta-analysis systematically reviewed risk factors, most included studies were cross-sectional in design, making it difficult to establish causal relationships; furthermore, some factors (e.g., poor sitting posture) lacked a standardized definition, compromising the comparability of results.

Recent region-specific studies have identified additional risk factors that expand our understanding of AIS etiology. A cross-sectional study from Italy further identified high-risk sports (e.g., gymnastics, dance) and postural disorders as significant factors associated with AIS, with adjusted ORs of 1.83 and 1.67, respectively (30). However, since the sample was drawn solely from the Province of Palermo, the generalizability of these findings is limited and requires validation in different populations. In China, a large-scale study in Gansu Province identified low BMI (OR = 0.91, 95% CI: 0.86–0.95), altitude of habitation (1,600–3,321 m, OR = 0.35–0.39), family medical history (OR = 1.66, 95% CI: 1.08–2.49), and shoulders of unequal height (OR = 1.45, 95% CI: 1.06–1.99) as independent factors associated with suspected scoliosis (25). Notably, the protective effect of higher altitude in this study is intriguing and warrants further investigation into the potential roles of environmental factors such as oxygen concentration and ultraviolet exposure. Another large epidemiological study in Dali, southwestern China, which included 139,922 children from 18 ethnic groups, found that age, gender, height, BMI, altitude, latitude, ethnicity, and county of residence were all significant influencing variables for suspected scoliosis (23). The finding of significant prevalence disparities among different ethnic groups suggests a potential genetic component that merits further exploration. Additionally, a study in Yunnan Province reported that the peak prevalence of idiopathic scoliosis occurred at age 13, and thoracolumbar curvature was the most common type among single-curvature cases (24). A cross-sectional study from Palestine identified low physical activity as an independent predictor of AIS (OR = 7.22, 95% CI: 1.64–31.79), along with significant associations with underweight and timing of the first menstrual cycle (28). These diverse findings across different populations and geographical settings underscore the multifactorial nature of AIS and highlight the need for region-specific prevention strategies.

### 3.4 Curve characteristics

Among diagnosed scoliosis cases, mild curves (Cobb angle 10°–19°) accounted for the highest proportion, at 86.65%; moderate curves (20°–39°) accounted for 12.47%; and severe curves (≥40°) accounted for only 0.89% (1). This distribution suggests that the majority of patients can be managed with conservative treatment, but the follow-up management strategies for mild curves still lack unified standards, potentially leading to either overtreatment or insufficient follow-up for some patients. Regarding curve type, single thoracolumbar curves were the most common (36.41%), followed by single thoracic curves (26.87%) and single lumbar curves (19.20%); idiopathic scoliosis accounted for 93.60% of all cases (1). Treatment selection varies among different curve types, but the prognostic differences between types have not been sufficiently analyzed, making it difficult to fully support individualized treatment planning.

## 4 Screening strategies

### 4.1 Screening necessity and controversies

The core objective of school-based scoliosis screening is to detect moderate curves amenable to bracing at an early stage, preventing progression to a severity level requiring surgical intervention (31). However, the clinical value of this screening model has long been debated. Opponents argue that school screening may lead to unnecessary radiation exposure, family anxiety, and a waste of medical resources (32, 33). In its 2018 recommendation statement, the U.S. Preventive Services Task Force assigned school-based scoliosis screening an “I” (insufficient evidence) grade, neither recommending for nor against it (34). This stance reflects the limitations of current screening research, particularly the lack of long-term cost-effectiveness analysis data. In contrast, the International Society on Scoliosis Orthopaedic and Rehabilitation Treatment and the Scoliosis Research Society explicitly support school screening under specific conditions (35, 36). Furthermore, a study has demonstrated that discontinuing school screening leads to diagnostic delays, with the average maximum Cobb angle at initial presentation increasing from 20° to 23° (37). While this finding underscores the early detection value of screening, the goal of early intervention may still not be achieved if the screening process is not standardized or screeners lack professional competence.

### 4.2 Screening methods and effectiveness evaluation

The current standard screening workflow primarily includes visual inspection, the Adam's forward bend test, and measurement of the angle of trunk rotation (ATR) using a scoliometer (38, 39). An ATR ≥5° is commonly used as the referral threshold for radiographic examination. However, relying solely on visual inspection during the Adam's test has low sensitivity and may lead to missed diagnoses. A 10-year school screening study from Croatia showed that the positive rate of the forward bend test rose from 4.9% to 5.8%, with a positive predictive value of 84.7% (40). This study benefits from a long follow-up period and high data credibility, but the screening subjects were limited to local students, and inter-operator consistency was not examined, making the results difficult to directly generalize.

Table 1 summarizes the characteristics of these novel home-based screening tools. The development of novel home-based screening tools has created possibilities for early assessment at home. For instance, a revised version of the “Scolioscope,” a simple home self-screening tool, achieved a sensitivity and specificity of 94% when operated by parents, using a Cobb angle >10° as the gold standard (41). This tool is simple and reasonably accurate but remains in the validation phase, lacking standardized operating procedures, and its long-term stability awaits observation. Another study developed a Scoliosis Tele-Screening Test (STS-Test), which also demonstrated high accuracy (83.51%) and specificity (98.87%), enabling parents to preliminarily assess their child's scoliosis risk without professional guidance (42). The development of smartphone applications has opened new avenues for home screening, with research confirming that non-professionals can reliably measure ATR using a smartphone inclinometer app (43). However, the popularization of such remote tools is constrained by family economic conditions and parental operational ability, making it difficult to reach all children and adolescents.

**Table 1:** Comparison of three screening methods for adolescent idiopathic scoliosis.

Comparison dimension	Scolioscope (Holleman et al., 2025) (41)	STS-test (Yilmaz et al., 2023) (42)	Smartphone app screening (Beauséjour et al., 2022) (43)
Principle	Household self-testing device based on scoliometer for ATR measurement	Trunk posture assessment test under remote video guidance	ATR measurement via smartphone inclinometer application
Sensitivity	94%	83.51%	Not reported (only reliability documented)
Specificity	94%	98.87%	Not reported
Operator requirements	Operable by parents after brief training	Self-operation by parents with remote guidance	Operation by parents following standardized training
Advantages	High accuracy, simple operation, suitable for family preliminary screening	Extremely high accuracy, remote screening available, no extra equipment required	High equipment popularity, low cost, convenient operation
Limitations	Still under verification, absence of standardized operating procedures	Dependent on network environment and parents’ comprehension capacity	Accuracy strongly relies on standardized training; large-sample verification is lacking

### 4.3 Differences in screening policies across countries

Globally, scoliosis screening policies for children and adolescents exhibit significant geographical variation (44). In the United States, policies vary by state, with some states implementing routine school screening while others do not. Canada, the United Kingdom, and Australia explicitly do not recommend routine school screening. Asian countries such as Japan, Singapore, and China generally conduct school screening. In Europe, only a few countries support school screening (40, 45). These differences are closely related to national healthcare resource allocation, health policy orientation, and disease prevalence characteristics. However, comparative effectiveness studies on different screening policies are lacking, making it difficult to determine the optimal model. In 2021, China issued the “Clinical Practice Guideline and Pathway for Adolescent Scoliosis Screening in China,” and in 2024, an updated version for the public was released, proposing a two-step strategy combining home screening and school screening (46). This strategy aligns with the Chinese context, but its implementation effect requires long-term monitoring, and varying degrees of execution across regions may affect the consistency of screening outcomes.

## 5 Conservative treatment

### 5.1 Exercise therapy

#### 5.1.1 Physiotherapeutic scoliosis-specific exercises (PSSE)

Physiotherapeutic scoliosis-specific exercises (PSSE) are individualized exercise intervention programs designed for scoliosis, implemented under the guidance of rehabilitation professionals in a clinical setting. They aim to reduce spinal deformity through three-dimensional self-correction, postural stabilization training, and integration into activities of daily living (47). It is important to note that these PSSE methods are not interchangeable; each has specific indications based on curve characteristics and patient presentation. The Schroth method, which emphasizes three-dimensional sensorimotor training and rotational breathing, is particularly indicated for patients with larger curves (typically >40°) with apical vertebral rotation and trunk decompensation (48, 50). BSPTS, which evolved from the Schroth tradition but incorporates modifications adapted to modern bracing principles (particularly the Chêneau-type brace), shares similar indications while offering a more streamlined exercise protocol. In contrast, SEAS—which is based on principles derived from the Lyon approach—emphasizes active self-correction and postural stabilization through task-oriented exercises and is suitable for a broader range of curve magnitudes, including milder curves (15°–30°), and places particular emphasis on patient autonomy and integration of corrective postures into daily activities (13, 48). Among these, the Schroth method is the most widely used and extensively studied. Multiple randomized controlled trials and meta-analyses have confirmed that Schroth exercises can effectively reduce the Cobb angle and ATR and improve quality of life (13, 49, 50). One randomized controlled trial showed that a 12-month supervised Schroth program combined with bracing was significantly superior to bracing alone in improving Cobb angle, ATR, and SRS-22 scores (49). Although this study has a rigorous design, the follow-up was only 12 months, making it difficult to assess long-term efficacy, and the limited sample size may affect the stability of the conclusions. A network meta-analysis comparing six PSSE methods indicated that SEAS was the most effective in reducing the Cobb angle, while active self-correction and Schroth performed best in improving ATR and quality of life (13). However, the studies included in this analysis were of variable quality, with several having small sample sizes and short follow-up periods, and a lack of direct head-to-head comparisons between different methods necessitates caution when evaluating the merits of each approach.

#### 5.1.2 Core stabilization training and other methods

Core stabilization training aims to enhance the strength and endurance of the muscles surrounding the spine, increasing spinal stability to help control curve progression. A comparative study demonstrated that Schroth training, spiral stabilization training, and core stabilization training all improved the Cobb angle and ATR, but the magnitude of improvement was significantly greater with the former two than with core stabilization training (51). This study clarified the efficacy differences among methods but did not explore the impact of individual patient characteristics (e.g., curve type, severity) on treatment outcomes, which limits individualized program development; moreover, the short follow-up period leaves long-term effects unclear. No significant differences were observed among the three groups in pain relief and quality of life improvement, suggesting that although core stabilization training is slightly weaker in deformity correction, it still holds value in improving subjective symptoms. Another randomized controlled trial focusing on patients with mild AIS confirmed that Schroth exercises were superior to core stabilization exercises in improving Cobb angle, spinal mobility, and quality of life, whereas the latter had a greater advantage in enhancing peripheral muscle strength (15). This study focused on mild cases, so the conclusions are difficult to extrapolate to moderate or severe populations, and the effectiveness of combining the two training modalities remains unclear and requires further investigation.

An additional factor that warrants consideration is the influence of chronological and physiological age on the responsiveness to different exercise modalities. Core stabilization training, which primarily targets the endurance and strength of type I and type II muscle fibers surrounding the spine, may be less effective in prepubertal and early pubertal children, particularly those under the age of 14. One proposed mechanism relates to the developmental maturation of hepatic enzymes involved in anaerobic glycolysis, which are essential for type II muscle fiber conditioning and typically do not reach adult levels until after the onset of puberty. This physiological limitation may explain why core stabilization exercises tend to yield more modest improvements in curve correction compared to PSSE in younger patients, as observed in some comparative studies (15, 51). Conversely, PSSE methods such as Schroth and SEAS, which emphasize sensorimotor integration, breathing mechanics, and active self-correction, may engage neuromuscular pathways that are less dependent on anaerobic metabolic capacity and may therefore be more suitable for younger patients. However, direct evidence linking these age-related physiological changes to specific exercise outcomes remains sparse, and further studies are needed to determine the optimal exercise prescription for patients at different developmental stages.

### 5.2 Brace treatment

Brace treatment for adolescent idiopathic scoliosis can be broadly categorized into three main types based on design philosophy, wearing schedule, and biomechanical action: rigid braces, soft (or dynamic) braces, and nighttime braces. Rigid braces, such as the Boston brace, Milwaukee brace, and Chêneau-type braces, are the most commonly used and function by applying three-point or four-point pressure systems to the trunk to correct the spinal curvature in the coronal, sagittal, and transverse planes. They are typically prescribed for full-time wear (16–23 h per day) and are indicated for patients with moderate curves (generally 25°–45°) who have significant remaining growth potential. Soft braces, such as the SpineCor system, utilize elastic bands and a pelvic base to guide spinal alignment through dynamic correction and active postural re-education. These braces are less rigid and may offer greater comfort and compliance, but their efficacy compared to rigid braces remains debated. Nighttime braces, including the Charleston bending brace and the Providence brace, are designed for wear only during sleep and apply hypercorrective forces in the supine position, leveraging the absence of gravitational loads to achieve curve correction. They are primarily indicated for patients with single, flexible thoracolumbar or lumbar curves. The choice of brace type depends on multiple factors including curve magnitude, curve location, skeletal maturity, patient compliance, and the availability of orthotic expertise, and should be individualized for each patient (14).

Given the diversity of brace types, bracing remains a standard conservative treatment for moderate to severe AIS (typically with a Cobb angle of 25°–45°). Its core function is to correct the spinal curvature through external forces, preventing progression. The BrAIST trial demonstrated that the success rate of brace treatment (72%) was significantly higher than that of observation (48%), and a dose-response relationship was observed between brace-wearing time and treatment success (14). Although this trial provided strong evidence for bracing, the enrolled patients were limited to specific age groups and curve types, which somewhat limits the generalizability of the conclusions. The trial also did not fully consider the impact of compliance on efficacy; some patients’ decreased compliance due to discomfort may have reduced the treatment's effectiveness. These observations highlight that brace prescription should extend beyond curve magnitude to incorporate patient-specific factors such as trunk morphology, flexibility, and functional status, as a brace that achieves optimal in-brace correction in one patient may yield suboptimal results in another with a similar radiographic curve. A 5-year observational study from Germany reported that in-brace correction was the only variable significantly associated with a curve improvement of ≥6°, while actual wear time had no significant impact on the treatment outcome (52). This finding differs from the BrAIST trial results, possibly due to differences in study populations, brace types, and follow-up management models, further underscoring that the factors influencing bracing efficacy are complex and require comprehensive individual evaluation. A prospective cohort study explored the effect of intensive bracing management combined with PSSE for AIS patients with 40°–60° curves who refused surgery, reporting a per-protocol treatment success rate of 83%; multivariate regression analysis identified a smaller baseline curve, a higher Risser sign, and a higher in-brace correction rate as independent predictors (53). This study provides a new conservative treatment concept for patients with moderate-to-severe curves, but the small sample size and single-center design mean the reliability of the conclusions requires validation through multicenter, large-sample studies.

Beyond the evidence from individual studies, the integration of bracing with physiotherapeutic scoliosis-specific exercises represents a promising direction for optimizing conservative management. While the brace provides external corrective forces and passive spinal realignment during wear, PSSE can enhance active neuromuscular control, postural awareness, and self-correction strategies that may improve the correction achieved within the brace and help maintain gains during brace-weaning periods (53). Emerging evidence suggests that combined bracing and PSSE protocols may yield superior outcomes compared to bracing alone, particularly when the exercise program is tailored to complement the specific corrective action of the prescribed brace (49, 53). Therefore, an integrated approach that aligns orthotic management with therapeutic exercise—rather than viewing them as separate or competing modalities—may represent the optimal conservative treatment strategy for patients with moderate to severe AIS, though further high-quality comparative studies are needed to define the most effective combination protocols.

### 5.3 Combined application of exercise and bracing

Multiple studies have confirmed that the clinical effects of PSSE combined with bracing may be superior to bracing alone (49, 53). The recent CONTRAIS randomized controlled trial compared three interventions: nighttime bracing, PSSE, and physical activity alone. The results showed that although nighttime bracing was superior to the PSSE group in controlling trunk rotation angle (mean difference −2.0°), there were no significant differences among the three groups in final spinal appearance, quality of life, or treatment adherence (54). This trial suggests that differences in efficacy among conservative treatment options may be small, and treatment outcomes are closely related to individual patient characteristics; currently, robust criteria for precise patient stratification are lacking. Moreover, the follow-up period in this trial was short, preventing observation of recurrence after skeletal maturity; it also did not analyze the efficacy differences between different PSSE methods in combination with bracing, making it difficult to fully optimize the combined treatment strategy.

## 6 Biomechanical research and spinal growth

Biomechanical factors play a critical role in the pathogenesis, progression, and treatment of adolescent idiopathic scoliosis. Advances in computational modeling, particularly finite element analysis, have enabled researchers to simulate spinal mechanical behavior under various loading conditions and to investigate the asymmetric stress distribution that may drive curve progression. Concurrently, a growing body of evidence has highlighted the importance of spinal growth patterns and sagittal parameters in understanding disease trajectory and optimizing intervention timing. Furthermore, radiation-free surface topography techniques have emerged as promising tools for scoliosis screening and longitudinal follow-up. This section synthesizes recent findings across these three interconnected areas—finite element modeling, spinal growth and sagittal alignment, and surface topography analysis—to provide a biomechanical perspective on AIS that complements the clinical and epidemiological evidence presented in preceding sections.

### 6.1 Finite element analysis in scoliosis research

Finite element analysis (FEA) can accurately simulate the mechanical behavior of the spine under various loading conditions, providing theoretical support for elucidating the biomechanical mechanisms of AIS and optimizing treatment strategies (16–18). One team constructed FEA models of the normal and AIS thoracolumbar spine and found that the AIS model exhibited higher primary range of motion, more pronounced segmental mobility asymmetry, and significantly greater mechanical loads on the intervertebral discs compared to the normal model (18). This model offers an important reference for understanding the mechanical abnormalities in AIS, but its construction simplified the roles of soft tissues such as paraspinal muscles and ligaments, thereby differing from the physiological state, and its clinical translational value requires validation. Another modeling study suggested that the mid-thoracic spinal cord is at risk of injury during surgical correction, and different correction maneuvers have varying effects on nerve roots, providing a theoretical basis for surgical plan optimization (17). However, the model did not account for individual anatomical variations, limiting its direct applicability to patient-specific surgical decisions, and the simulated surgical procedures differ from real-world operations, necessitating further clinical corroboration. Another group proposed a high-precision modeling method combining CT and radiographic images, revealing a gravity-induced shift of the spine towards the concavity and asymmetric stress distribution, hypothesizing that this asymmetric loading may affect bone growth and development through the Hueter-Volkmann principle, thereby promoting curve progression (16). This modeling method improves precision, but due to its complexity and time-consuming nature, clinical dissemination is limited. Moreover, the causal relationship between asymmetric stress distribution and curve progression is solely a theoretical inference lacking *in vivo* experimental evidence.

### 6.2 Spinal growth and sagittal parameters

The growth and development of the spine are closely related to the progression of AIS. Clarifying growth patterns can inform disease prognosis and treatment timing. One study provided spinal growth multipliers based on peak height velocity age for predicting the final spinal length of patients (55). These multipliers offer a tool for quantifying spinal growth potential, but the study population was predominantly Caucasian, and ethnic differences may affect their applicability, requiring validation in Asian populations. Pelvic incidence (PI) is a core morphological parameter for assessing sagittal spinal balance. A longitudinal study of AIS patients showed that PI reaches its peak growth velocity at Risser stage 1 and continues to increase after skeletal maturity (Risser stage 5) (56). This study reveals the growth pattern of PI, but it did not analyze the association between PI changes and curve progression, making it difficult to use PI as a predictor of disease advancement. The follow-up period was also limited, and the trend of PI changes into adulthood and its impact on prognosis remain to be elucidated.

### 6.3 Surface topography analysis

Surface topography analysis is a radiation-free method for assessing scoliosis, aiding in screening and follow-up by quantifying back asymmetry (57, 58). Three-dimensional back shape analysis systems, such as rasterstereography, which are currently in common use, can objectively quantify back asymmetry while avoiding radiation exposure. However, their detection accuracy is affected by the environment and patient posture, and the high cost of equipment hinders their promotion in primary care settings. Researchers have developed an automated, non-invasive detection system based on depth sensors that can complete back shape analysis within 1.5 s, achieving an area under the curve (AUC) of 0.96 for predicting a Cobb angle >25° (59). This system features high speed and accuracy but is still in the laboratory validation stage and has not yet undergone large-scale clinical application, so its long-term stability and reliability need further observation. Furthermore, photogrammetry has been confirmed to effectively detect chest wall morphological changes in AIS patients, and these chest wall parameters correlate with the Cobb angle and pulmonary function (58). This method is simple and low-cost, but the accuracy of the results depends on the camera angle and operator standardization, and inter-rater consistency needs improvement. Moreover, the causal relationship between chest wall morphological changes and curve progression remains unclear, making it difficult to adopt as a core indicator for disease monitoring for the time being.

## 7 Artificial intelligence and machine learning applications

### 7.1 Prediction models

Machine learning techniques can integrate multi-dimensional clinical data to construct prediction models for AIS progression, offering precise references for individualized treatment decisions (19, 20, 60). A systematic review outlined the current application status of machine learning in AIS progression prediction, revealing both the advantages and limitations of this technology (19). One study used a random forest model to predict AIS curve progression, identifying the Cobb angle and spinal flexibility as key predictors, with a mean absolute error of 4.64° (60). Although this model achieved relatively high prediction accuracy, the set of predictors was limited, excluding intrinsic factors such as genetics and hormones. Additionally, it was constructed based on single-center data and lacks external validation, limiting its generalizability. Another study built a composite prediction model integrating global/regional radiographic data and clinical parameters, achieving an AUC of 0.84 for predicting progressive AIS curves, significantly outperforming traditional methods (61). This model benefits from a more comprehensive data source but is structurally complex, making it difficult to use at the grassroots level, and its poor interpretability makes it challenging for clinicians to understand the decision-making logic, thereby affecting its acceptance. Importantly, all AI-based prediction tools should be viewed as decision-support instruments rather than autonomous diagnostic or prognostic systems; their outputs must be interpreted in the context of the individual patient's clinical presentation, history, and physical examination findings by an experienced clinician.

### 7.2 Automated image analysis and explainability

Deep learning technology has been successfully applied to the automated analysis of spinal radiographs, reducing the manual measurement time for parameters such as the Cobb angle from several minutes to a few seconds, with good reliability of the results (62, 63). This offers possibilities for large-scale screening and disease monitoring but is highly dependent on high-quality imaging data; equipment limitations at the primary care level may restrict its accuracy. The development of explainable artificial intelligence (XAI) methods can help clinicians understand the basis of model decisions, thereby enhancing model acceptance (19, 64). However, most XAI techniques are still in their early stages, making it difficult to comprehensively analyze the logic of complex models. A lack of standardized evaluation metrics for explainability also makes comparisons across different studies challenging. More critically, most current AI studies lack clear clinical implementation guidelines and external validation data, meaning the translation from the laboratory to clinical practice still faces numerous obstacles (19). A systematic review clearly stated that while AI shows good potential in AIS image analysis, the supporting evidence base still needs to be strengthened, and more large-scale, multicenter validation studies are urgently needed to support its routine clinical application (65). At present, AI cannot replace the comprehensive clinical judgment of an experienced physician, nor can it account for the nuanced psychosocial and functional considerations that are integral to clinical decision-making in AIS management. Rather, AI should be positioned as a complementary tool that enhances efficiency and provides data-driven insights, while the ultimate treatment decisions remain grounded in clinical expertise and shared decision-making with patients and families.

## 8 Discussion and outlook

### 8.1 Main findings and clinical significance

This review integrates existing evidence, indicating that scoliosis in children and adolescents is a global public health issue. The global prevalence based on radiographic diagnosis is approximately 1.65%, with females having roughly twice the risk of males (1). Its risk factors encompass multiple dimensions, including genetics, lifestyle, and behavioral habits, with female sex, family history, poor sitting posture, and prolonged screen time identified as clear high-risk factors (1, 30). These findings provide a basis for targeted screening of high-risk populations, but current risk factor studies are primarily correlational, and causality remains unestablished, making it difficult to directly guide preventive measures. Early screening strategies are evolving from traditional school screening towards a diversified approach combining simple home-based self-screening tools and remote assessment (41, 42). While this can improve the convenience and accessibility of screening, the standardization and widespread adoption of these tools still need to be further promoted. In terms of conservative treatment, PSSE (especially Schroth and SEAS methods) and bracing can both effectively control curve progression, but the treatment plan should take into account individual characteristics and mental health (9, 13, 14, 54). Currently, the stratification basis for individualized treatment is insufficient, and unified clinical guidelines are still lacking. Biomechanical research (e.g., finite element analysis, surface topography analysis) and artificial intelligence technologies offer new perspectives for elucidating disease mechanisms and optimizing treatment decisions (16–18, 59), but the clinical translation of these technologies still faces many challenges and has not yet become routine practice.

### 8.2 Challenges and future directions

Although significant progress has been made in the field of scoliosis in children and adolescents, there are still many controversies and challenges. Future research should focus on the following directions.

First, advancing precision treatment and patient stratification. Existing studies report inconsistent conclusions regarding the efficacy of exercise therapy and bracing, which may be related to differences in curve severity, intervention intensity, and outcome measures. In this context, the role of physiological maturation in modulating treatment response deserves particular attention. The effectiveness of different conservative treatment modalities may vary considerably across developmental stages, partly due to age-related differences in neuromuscular maturation, metabolic capacity of skeletal muscle, and skeletal growth velocity. For instance, core stabilization training may be less effective in prepubertal children due to the delayed maturation of hepatic enzyme systems essential for anaerobic glycolysis, whereas PSSE methods that emphasize sensorimotor integration and breathing mechanics may engage neuromuscular pathways that are less dependent on anaerobic capacity and may therefore be more suitable for younger patients. Similarly, the timing of brace initiation relative to the peak height velocity is a well-established determinant of treatment success. These observations collectively suggest that treatment stratification should not be based solely on curve magnitude and type, but should also incorporate indicators of physiological maturation—such as chronological age, Risser sign, peak height velocity age, and possibly biomarkers of neuromuscular development—to better match patients with the most appropriate intervention. Future large-scale, multicenter, long-term follow-up randomized controlled trials are needed to compare the efficacy of different PSSE and bracing combination protocols, achieve precise stratification by combining biomarkers and clinical phenotypes, and establish unified efficacy evaluation criteria to enhance the comparability of studies (66). Furthermore, patient stratification should explicitly incorporate mental health indicators, as psychological distress—including anxiety, depression, and body image concerns—can significantly influence treatment adherence and satisfaction with conservative care. Routine integration of standardized psychological screening tools (e.g., SRS-22 psychological domain, Beck Depression Inventory, or scoliosis-specific body image scales) into clinical management pathways would enable early identification of at-risk patients and facilitate timely psychosocial interventions alongside physical treatment.

Second, strengthening the integration of mental health. AIS patients have a high incidence of mental health problems (e.g., anxiety, depression, body dysmorphic disorder), which affect treatment adherence and quality of life (9), yet current clinical management pathways do not routinely incorporate psychological assessment and intervention. Future work should formulate standardized mental health assessment processes, explore targeted psychological intervention measures, and organically integrate them with physical treatment to achieve comprehensive psychosomatic care. Crucially, psychological well-being—including body image satisfaction, self-esteem, and social functioning—should be monitored as an independent outcome domain throughout the life course, given its persistent impact on quality of life even after skeletal maturity.

Third, promoting radiation-free/low-radiation assessment technologies. Traditional radiographic assessment carries a cumulative risk of ionizing radiation. Radiation-free or low-radiation techniques such as 3D ultrasound, surface topography, and AI-assisted analysis have shown good application potential (41, 42, 59). Future efforts should strengthen clinical validation, optimize technical workflows, reduce costs, and promote their adoption at the primary care level to minimize patients’ radiation exposure to the greatest extent possible.

Fourth, facilitating the clinical translation of artificial intelligence. Current AI models perform well in screening, diagnosis, and prediction, but deficiencies in explainability, external validation, and clinical implementation guidelines hinder their integration into routine workflows (19, 65). It is essential to recognize that AI is designed to augment—not replace—clinical expertise. Future work must deepen explainability research, conduct multicenter external validation, develop standardized implementation guidelines, and enhance clinician training to improve model acceptance and application outcomes in clinical settings. Additionally, clinician-AI collaboration frameworks should be established to define clear roles for AI-generated outputs in the clinical decision-making process, ensuring that these tools serve as efficient assistants rather than autonomous decision-makers.

Fifth, establishing a life-cycle management model. Most studies have focused on treatment and follow-up during childhood and adolescence, with insufficient attention paid to the long-term prognosis of adult scoliosis patients who have not undergone surgery. These patients often suffer from chronic pain and functional limitations, with a markedly decreased quality of life (10, 67). In this context, particular attention should be paid to sagittal spinal alignment, which is increasingly recognized as a critical determinant of long-term prognosis in scoliosis patients. While current clinical decision-making in AIS has predominantly focused on coronal plane deformity (i.e., Cobb angle), emerging evidence from adult spinal deformity research suggests that sagittal imbalance may be more strongly associated with pain, functional impairment, and quality of life deterioration than coronal measures alone (10, 67). For adult patients with untreated or residual idiopathic scoliosis, progressive sagittal decompensation—manifested as increased pelvic tilt, reduced lumbar lordosis, or positive sagittal vertical axis—may drive the clinical course and exacerbate symptoms even in the absence of significant coronal curve progression. However, longitudinal data linking specific sagittal parameters to long-term outcomes in AIS patients from adolescence through adulthood are currently sparse. Future research should strengthen the long-term follow-up of adult patients, elucidate key prognostic factors such as curve severity, spinal mobility, sagittal alignment parameters, and perception of trunk cosmesis, and construct a management system covering the entire life cycle to effectively improve patients’ long-term outcomes. Crucially, psychological well-being—including body image satisfaction, self-esteem, and social functioning—should be monitored as an independent outcome domain throughout the life course, given its persistent impact on quality of life even after skeletal maturity.

## 9 Conclusion

Scoliosis in children and adolescents is a complex three-dimensional spinal deformity requiring multidisciplinary comprehensive management. Existing evidence supports the value of school-based screening conducted under specific conditions for early detection, and PSSE and bracing can effectively control curve progression; however, treatment plans need to be individualized and take mental health into account. Biomechanical research and artificial intelligence technologies provide new tools for mechanism elucidation and treatment decision optimization, but their clinical translation still requires substantial further advancement. Future efforts should focus on developing precision treatment strategies, popularizing radiation-free assessment technologies, and clinically translating artificial intelligence models, with the overarching goal of improving the long-term outcomes and quality of life of children and adolescents with scoliosis.

## Data Availability

The original contributions presented in the study are included in the article/Supplementary Material, further inquiries can be directed to the corresponding authors.
